# Multicenter Study of Nonadherence to Self-Injectable Biologic Therapy in Patients With Inflammatory Bowel Disease: Risk Factors and Outcomes

**DOI:** 10.1016/j.gastha.2023.01.016

**Published:** 2023-01-30

**Authors:** Lauren A. George, Erin Causey, Nisha B. Shah, James C. Slaughter, Jonah Jerabek, Autumn D. Zuckerman, Susan Chhen, Elizabeth Scoville, Robin Dalal, Dawn B. Beaulieu, Baldeep Pabla, David A. Schwartz, Raymond K. Cross, Byron P. Vaughn, Sara Horst

**Affiliations:** 1 Division of Gastroenterology & Hepatology, University of Maryland School of Medicine, Baltimore, Maryland; 2 Vanderbilt Specialty Pharmacy, Vanderbilt University Medical Center, Nashville, Tennessee; 3 Division of Gastroenterology, Hepatology & Nutrition, Vanderbilt University Medical Center; 4 Division of Gastroenterology, Hepatology, & Nutrition, University of Minnesota, Minneapolis, Minnesota; 5 Fairview Specialty Pharmacy, Minneapolis, Minnesota

**Keywords:** Adherence, Crohn’s Disease, Ulcerative Colitis, Biologic Therapy

## Abstract

**Background and Aims:**

This study aimed to evaluate adherence to subcutaneous biologic therapy and impact of nonadherence including risk factors and outcomes in academic centers with integrated specialty pharmacies for patients with inflammatory bowel disease (IBD).

**Methods:**

This was a multicenter, retrospective cohort analysis of patients aged ≥18 years receiving care in 3 tertiary care outpatient IBD clinics with integrated specialty pharmacies. Subjects were prescribed injectable anti-TNF therapy (adalimumab, certolizumab, golimumab) or anti-IL 12/23 therapy (ustekinumab) with at least 3 consecutive prescription claims. The primary outcomes were medication possession ratio (MPR), percent achieving optimal adherence (MPR > 0.86); in addition, we sought to verify a prior risk factor model including smoking status, narcotic use, psychiatric history, and prior biologic use. Secondary outcomes included emergency department visits (ED) and IBD-related hospitalizations. Statistical analysis was performed using Wilcox rank sum test, Pearson’s Chi-squared test, and logistic regression model as an unordered, factor variable to flexibly estimate the probabilities of adherence.

**Results:**

Six hundred eight subjects were included. Overall median MPR was 0.95 (interquartile range 0.47, 1) and adherence was 68%–70%. When the number of risk factors for nonadherence increased, the likelihood of nonadherence increased (*P* < .05). In unadjusted and adjusted analysis, nonadherence increased the likelihood of ED visits [rate ratio 1.45 (95% confidence interval 1.05, 1.97)] and hospitalizations [rate ratio 1.60 (95% confidence interval 1.16, 2.10)].

**Conclusion:**

Academic centers with integrated pharmacies had high adherence. Prior risk factors for nonadherence remained significant in this multicenter model. Nonadherence was associated with higher likelihood of hospitalizations and ED visits.

## Introduction

Inflammatory bowel disease (IBD) is a chronic inflammatory disease of the gastrointestinal tract that includes Crohn’s disease (CD) and ulcerative colitis (UC).^1^ Disease exacerbation is often associated with emergency department (ED) visits, hospitalizations, change in maintenance medication, and steroid use to alleviate symptoms. Prolonged inflammation due to inadequately controlled disease can cause irreversible damage and complications; thus, induction and maintenance of disease remission is critical to avoid negative clinical outcomes and increased healthcare costs. Current treatment options for moderate to severe IBD include self-injectable biologic medications, including anti-TNF therapies (ie, adalimumab, certolizumab, and golimumab) and an interleukin 12/23 inhibitor (ustekinumab).^2^, ^3^, ^4^, ^5^ Maintaining optimal adherence is imperative to achieving durable clinical benefit. Nonadherence to medications increases healthcare costs, risk of disease flare, and antidrug antibody formation for anti-TNF therapies.^6^^,^^7^

Accurate measurement of adherence is essential to developing strategies to improve adherence; therefore, objective measures of adherence, such as medication possession ratio (MPR) are important and often used in pharmacy claims data.^8^ A large nationally representative claims database study of patients prescribed self-injectable anti-TNF medications found that patients with an MPR >0.86 for adalimumab and 0.87 for certolizumab had significantly lower risk of disease flare.^9^ This threshold can therefore be used to identify nonadherent patients.

Identifying patients at risk for nonadherence is important to develop strategies to improve adherence. Prior studies suggest risk factors for nonadherence to biologic therapy include younger age, prior nonadherence, psychiatric history, smoking, narcotic use, or noncommercial health insurance.^9^, ^10^, ^11^, ^12^, ^13^, ^14^, ^15^, ^16^, ^17^ Previous analysis evaluated risk factors for nonadherence (MPR < 0.86) to self-injectable biologic medications. This study found that, in a multivariable regression model, Medicaid insurance status and Crohn’s disease increased nonadherence. Also, several easily clinical identifiable factors in univariate analysis were significant (specifically smoking, narcotic use, psychiatric history, and prior biologic therapy). When the number of these risk factors for a particular patient increased, the likelihood of nonadherence significantly increased.^18^ As this was a single-centered study with limited demographic diversity, the generalizability of these findings was limited. Therefore, the aim of this analysis was to evaluate adherence across several academic centers with integrated specialty pharmacies, to assess if previously identified risk factors for nonadherence remained significant across several diverse IBD centers and to evaluate outcomes associated with nonadherence.

## Methods

This was a multicenter, retrospective cohort analysis of patients aged ≥18 years receiving care at 1 of 3 tertiary care outpatient clinics (Vanderbilt University Medical Center, University of Maryland, and University of Minnesota) for either active CD or UC. Study period was from January 1, 2018 through December 31, 2019 for center 1 and March 2018 to March 2019 for center 2 and August 2018 to July 2020 for center 3 (available data from the specialty pharmacies were slightly different secondary to differing reporting strategies per center at the time of data collection). Patients prescribed a subcutaneous anti-TNF therapy (adalimumab, certolizumab, golimumab) or anti-IL 12/23 therapy (ustekinumab) and receiving medication through the center’s integrated specialty pharmacy (to permit access to pharmacy claims data) were included. Patients with less than 3 specialty prescription claims or a change in pharmacy during the study period were excluded, as these confounders would limit the ability to accurately calculate medication adherence.^8^ Patients were followed until end of study date, medication was stopped, or loss of follow-up occurred. Patients who changed to a new biologic only had the first biologic evaluated.

Baseline disease characteristics, surgical history, previous biologic therapy, current smoking status, concomitant therapy for IBD, and active narcotic prescription at the time of biologic initiation were extracted from the electronic medical record (EMR). Insurance status was assessed at baseline. Psychiatric comorbidity was defined as a documented diagnosis in the EMR (including depressive disorders, anxiety disorders, bipolar disorders, and/or psychotic disorders). Prescription claims data extracted from the specialty pharmacy database were analyzed to identify patient-specific medication dispensing dates and payment methods. Data were managed using Research Electronic Data Capture (REDCap).^19^^,^^20^ IBD-related emergency department visits and hospitalizations in follow-up were extracted from the EMR.

The primary outcome of medication adherence across several academic centers with integrated specialty pharmacies was assessed using MPR, calculated as the total sum of days’ supply for each medication refill divided by the number of days in the observation period and was calculated from fill dates using the same formula for all centers.^9^ This adherence measurement is accurate regardless of dosing strategies for adalimumab, certolizumab, and golimumab as it accounts for prescription required days’ supply. For ustekinumab, since the prescribed day supply often exceeds the 30-day supply limit mandated by some third-party benefit managers at the time of a prescription claim, we used the prescribed day supply as noted in the EMR prescription for the denominator. The MPR calculation was capped at one. We defined nonadherence as MPR <0.86 based on prior research.^9^

Secondary outcomes included determination of the effect of prior identified risk factors (smoking, narcotic use, psychiatric history, and prior biologic use) on nonadherence in a multicenter cohort. Other secondary outcomes included the impact of nonadherence on number of ED visits and hospitalizations. Unadjusted association between adherence or disease status and prespecified risk factors were tested using either the Wilcox rank sum test (continuous variables) or Pearson’s Chi-squared test (categorical variables). Unadjusted and adjusted odds ratios with corresponding 95% confidence intervals (CIs) were estimated using univariable and multivariable logistic regression, respectively. Initial risk factors were prespecified and included in the models regardless of statistical significance. All relevant risk factors were reduced to a score (0–4) by summing 4 binary risk factors: prior biologic use, current narcotic use, smoking, and any psychiatric history. These variables were chosen as they had significance in prior literature and our prior work. The risk score was then included in a logistic regression model as an unordered, factor variable to flexibly estimate the probabilities of adherence for subjects with risk scores of 0, 1, 2, 3, or 4. All statistical analyses were performed using the R statistical package.

### Ethical Considerations

This was a retrospective study with no significant ethical considerations.

## Results

Six hundred eight patients were included (center 1, originator center: n = 460, center 2: n = 81, and center 3: n = 67). Overall median days followed was 903 days (interquartile range 187, 3900 days) and did not differ between centers. Table 1 summarizes patient characteristics. Generally, patients had similar characteristics across the 3 centers; however, centers 2 and 3 had more non-White patients and patients with Medicaid insurance. Center 3 had more patients with UC and less patients who had previous surgery, been on prior biologics, required narcotics, or had perianal or fistulizing disease, suggesting a patient population with less severe disease.

**Table 1:** Demographics and Clinical Characteristics of Patients With Inflammatory Bowel Disease on Subcutaneous Biologic Therapy Across IBD Centers

	Center 1 n = 460	Center 2 n = 81	Center 3 n = 67	*P* value
Median follow up, d (range)	921 (232, 1414)	1524 (114, 3900)	730 (159, 3430)	NS
Median age (range)	37 (30, 48)	35 (27, 44)	33 (22, 43)	NS
Median dis duration, y (range)	9 (4, 16)	9 (4, 17)	5 (1, 9.5)	<.05
UC	15% (67)	14% (11)	25% (17)	<.05
Female	62% (283)	56% (45)	52% (35)	NS
Caucasian	92% (423)	84% (68)	69% (46)	<.05
Smoker	17% (76)	10% (8)	9% (6)	<.05
Insurance				<.05
Commercial	74% (340)	74% (60)	69% (46)	
Medicare	21% (97)	5% (4)	3% (2)	
Medicaid	4% (20)	21% (17)	28% (19)	
None	1% (3)	0	0	
Psychiatric history	38% (150)	41% (29)	26% (13)	NS
Biologic therapy history	59% (271)	63% (51)	42% (28)	NS
Crohn's disease	n = 393	n = 74	n = 69	NS
Perianal disease	38% (150)	41% (29)	26% (13)	
Fistulizing disease	54% (212)	63% (51)	42% (28)	
Past surgical history	54% (216)	63% (47)	34% (24)	

Seventy percent of the population was adherent (MPR > 0.86). The median MPR was high at 0.95 (interquartile range 0.47, 1). MPR and adherence rates were similar between centers as detailed in Table 2. Univariate analysis revealed the following were associated with nonadherence: female sex, noncommercial health insurance, and positive smoking status. Disease types in CD (perianal disease or fistulizing disease) were not significant (Table 3).

**Table 2:** Medication Possession Ratio and Adherence Rates of Patients With Inflammatory Bowel Disease on Subcutaneous Biologic Therapy Across IBD Centers

	Center 1 n = 460	Center 2 n = 81	Center 3 n = 67	*P* value
MPR^a^, mean (±SD)	0.89 ± 0.13	0.88 ± 0.16	0.90 ± 0.10	NS
Adherence (MPR > 0.86)	70% (323)	68% (55)	69% (46)	NS

a Medication Possession Ratio.

**Table 3:** Univariate Analysis for Risk Factors for Nonadherence to Self-Injectable Biologic Medications in Patients With IBD

	Nonadherent (MPR < 0.86) n = 184	Adherent (MPR > 0.86) n = 424	*P* value
Age, median (IQR range)	36 (27, 45)	37 (29, 48)	.9
UC	14% (26)	13% (55)	.33
Female	67% (124)	56% (239)	<.05
Caucasian	91% (165)	90% (381)	.76
Smoker	20% (36)	13% (54)	<.05
Narcotic use	28% (51)	21% (90)	.08
Insurance			<.05
Commercial	67% (124)	76% (322)	
Medicare	17% (31)	17% (72)	
Medicaid	15% (28)	7% (28)	
None	1% (1)	<1% (2)	
Psychiatric history	53% (96)	47% (197)	.17
Biologic therapy history	63% (116)	55% (234)	.07
Crohn's disease			
Perianal disease	36% (59)	38% (133)	.75
Fistulizing disease	54% (88)	48% (170)	.21

**Figure 1:**
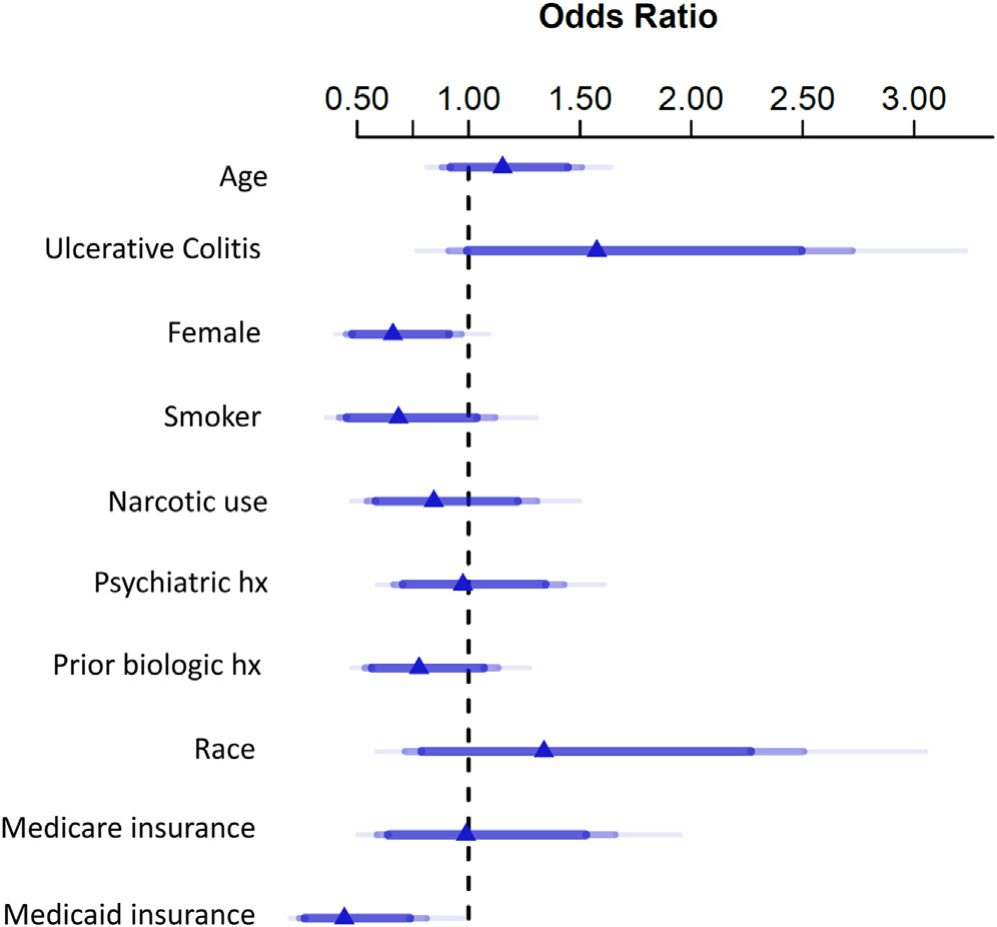
Multivariable regression analysis for risk factors for adherence in all patients with inflammatory bowel disease from 3 tertiary care IBD centers started on a self-injectable biologic medication.

**Figure 2:**
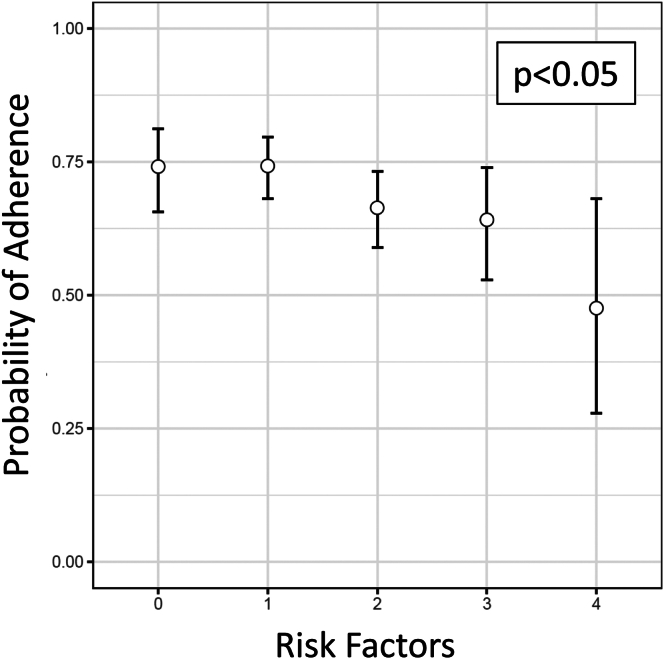
The overall probability of nonadherence (MPR < 0.86) for patients started on a self-injectable biologic medication from 3 tertiary care IBD centers significantly increases as the number of risk factors (prior biologic use, smoking, narcotic use, and psychiatric history) increase.

In multivariable logistic regression, patients with Medicaid insurance status had an increased risk of nonadherence (Figure 1). When the number of risk factors for nonadherence increased, the likelihood of nonadherence increased (*P* < .05) (Figure 2). Adherence (MPR > 0.86) was 74% if patients had 0–1 risk factors, decreasing to 66% when 2 risk factors were present, 64% when 3 risk factors were present, and only 48% if 4 risk factors were present (*P* < .05).

**Figure 3:**
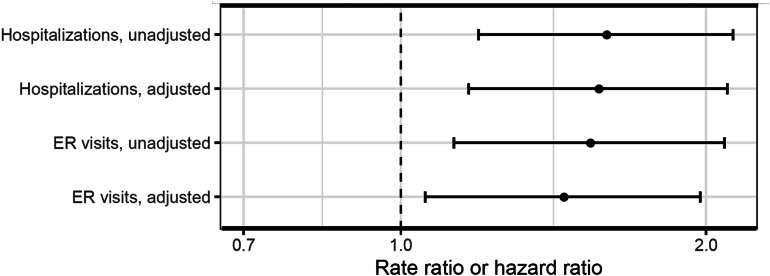
The overall likelihood of ED visits and hospitalizations increases if patients were nonadherent to self-injectable biologic medication (MPR < 0.86).

Twenty one percent (133/608) of patients had an IBD-related ED visit during the study period. Nonadherent patients were more likely to require IBD-related ED visits (27%) than adherent patients (20%). Figure 3 presents the unadjusted and adjusted rate ratios for ED visits and hospitalizations. In unadjusted analysis, nonadherence to self-injectable biologic medications increased the risk of ED visits (rate ratio 1.53, 95% confidence interval [CI] 1.1, 2.09). This remained significant when adjusting for age, sex, race, and disease type (rate ratio of 1.45, 95% CI 1.05, 1.97). Twenty three percent (141/608) of patients had IBD-related hospitalizations. Nonadherent patients were hospitalized more often (28%) compared to adherent patients (21%). In unadjusted analysis, nonadherence to self-injectable biologic medications increased the likelihood of hospitalization (rate ratio 1.59, 95% CI 1.19, 2.13). This remained significant when adjusting for age, sex, race, and disease type (rate ratio of 1.60, 95% CI 1.16, 2.10).

## Discussion

We found high adherence rates (70%) and high median MPR (0.95) for IBD patients on self-injectable biologic medications. This was similar across all centers, geographic regions, and patient demographics. Nonadherence significantly increased the risk of ED visits and hospitalizations. These findings highlight the impact of biologic adherence on direct patient outcomes and healthcare costs. We additionally validated previously identified risk factors in a multicentered cohort. Patients with increasing number of nonadherence risk factors had substantially lower adherence. Those with 4 risk factors had less than 50% adherence.

Adherence rates to self-injectable biologic medications for patients in this cohort were significantly higher than nationally reported adherence rates in IBD patients.^9^ Prior work has shown that integrated health systems specialty pharmacies such as those used in this cohort have demonstrated high rates of adherence across several chronic disease states including rheumatoid arthritis, multiple sclerosis, pulmonary arterial hypertension, and human immunodeficiency virus.^21^, ^22^, ^23^, ^24^, ^25^ Our findings add additional support to the benefit of this integrated specialty pharmacy team model, which may improve adherence through close co-management with the clinical IBD team. Health system specialty pharmacy teams perform patient outreach on a regular basis to assess for medication-related issues and try to improve adherence. The specialty pharmacy team also manages navigation of the insurance approval process which can be a very difficult aspect of continuing biologic therapy for patients.^26^ It is possible that other areas of support from tertiary care centers for inflammatory bowel disease could contribute to high adherence for patients.

However, even within our study with high adherence, 30% of patients did not meet the adherence threshold of MPR >0.86. Understanding risk factors for nonadherence can help inform patient-centered care delivery models for patients on subcutaneous biologic medications. A systematic review showed risk factors for nonadherence to anti-TNF therapy include female sex, smoking, anxiety, and moodiness.^17^ Also, a recent study from the national Manitoba cohort with prospective data collection showed that presence of anxiety and depression increases the likelihood of anti-TNF discontinuation.^10^ Our findings supplement prior work and further highlight the importance of an individual’s psychiatric history when selecting the appropriate IBD management strategy. Patients who have a high probability of nonadherence or discontinuation from biologic therapy may need closer monitoring and aggressive mental health support. In addition to psychiatric history, we found other patient factors such as smoking, narcotic use, and prior biologic use increases nonadherence. Other studies suggest these factors play a role in poor IBD outcomes in general. For instance, smoking is independently associated with poor IBD outcomes.^27^ Narcotic use and psychiatric disorders are associated with higher healthcare utilization.^28^ Prior biologic use may increase the likelihood of subsequent medication nonresponse and therefore less patient self-efficacy to stay adherent.^29^, ^30^, ^31^ Factors that may be out of a patient’s control, such as Medicaid insurance status, have been associated with nonadherence.^32^ These patients may have more restricted access to care and economic limitations.^33^ It is unlikely that any single factor is itself causal to nonadherence but rather reflects a complex psychosocial environment that can lead to nonadherence. Our multicenter study confirms there is a synergistic or cumulative effect of these factors resulting in nonadherence.

Our findings have important considerations for the cost of care for IBD patients on self-injectable biologic medications. We showed that nonadherence significantly increased the likelihood of IBD-related ED visits and hospitalizations. A recent study looking at the healthcare costs associated with patients with IBD found that patients with one ED visit had costs more than twice as high as patient without ED visits.^34^ Improving medication adherence for moderate to severe IBD is an easily definable target for healthcare systems to improve cost and enhance clinical outcomes. The high adherence data in this study lend support to the patient-centered multidisciplinary care. Other data have shown that integrated care models for patients with IBD can improve clinical outcomes including decreasing ED visits and hospitalizations.^35^^,^^36^

Strengths of this study include a multicenter approach with significant diversity in geographic location, race, and insurance status to allow for validation of our previous work. In addition, we used an objective marker of adherence across centers. However, there is limitation to using MPR, as it does measures medication acquisition and not direct administration. However, it is unlikely for a patient to continue obtaining a self-injectable biologic medication over more than a 6-month period without administering the medication consistently. MPR could not be assessed by patients outside of academic center specialty pharmacy as access to adherence data is not freely given by national contracting specialty pharmacies. Limiting to 3 or more claims to ensure stability on medication could miss those who would be most at risk for nonadherence. We also were limited to EHR data which may have missed some clinical outcomes including ED visits external to the related hospital system. We also did not have data regarding health literacy, education status, or specific reason for nonadherence such as disease activity at time of missed doses. Our study period did not allow for evaluation of newer mechanisms such as oral small molecule medication.

## Conclusion

In conclusion, our study shows high adherence rates for patients receiving self-injectable biologic medications who fill from an IBD center specialty pharmacy, exceeding previously reported national adherence rates. We validated a model demonstrating that increasing identifiable risk factors (smoking, narcotic use, psychiatric history, and prior biologic use) increases the likelihood of nonadherence. Patients with Medicaid insurance remain at a higher risk of nonadherence in this multicenter model. Nonadherence increases the probability of IBD-related ED visits and hospitalizations. All healthcare industry stakeholders including healthcare systems, manufacturers, and third-party benefit providers need to understand the importance of improving patient adherence. Decreasing barriers to self-injectable medication acquisition, increasing direct patient interaction with integrated pharmacy teams, and comprehensive patient education are a start to improving patient adherence. In addition, we propose that enhanced care pathways for patients with risk factors for nonadherence would improve adherence and outcomes.

## References

[1] Chang J.T. (2020). Pathophysiology of inflammatory bowel diseases. N Engl J Med.

[2] Feuerstein J.D., Isaacs K.L., Schneider Y. (2020). AGA clinical practice guidelines on the management of moderate to severe ulcerative colitis. Gastroenterology.

[3] Berg D.R., Colombel J.F., Ungaro R. (2019). The role of early biologic therapy in inflammatory bowel disease. Inflamm Bowel Dis.

[4] Lichtenstein G.R., Loftus E.V., Isaacs K.L. (2018). ACG clinical guideline: management of Crohn's disease in adults. Am J Gastroenterol.

[5] Hanauer S.B., Sandborn W.J., Feagan B.G. (2020). IM-UNITI: three-year efficacy, safety, and immunogenicity of ustekinumab treatment of Crohn's disease. J Crohns Colitis.

[6] Herman M.L., Kane S.V. (2015). Treatment nonadherence in inflammatory bowel disease: identification, scope, and management strategies. Inflamm Bowel Dis.

[7] Baert F., Noman M., Vermeire S. (2003). Influence of immunogenicity on the long-term efficacy of infliximab in Crohn's disease. N Engl J Med.

[8] Andrade S.E., Kahler K.H., Frech F. (2006). Methods for evaluation of medication adherence and persistence using automated databases. Pharmacoepidemiol Drug Saf.

[9] Govani S.M., Noureldin M., Higgins P.D.R. (2018). Defining an optimal adherence threshold for patients taking subcutaneous anti-TNFs for inflammatory bowel diseases. Am J Gastroenterol.

[10] Dolovich C., Bernstein C.N., Singh H. (2020). Anxiety and depression leads to anti-tumor necrosis factor discontinuation in inflammatory bowel disease. Clin Gastroenterol Hepatol.

[11] Lopez A., Billioud V., Peyrin-Biroulet C. (2013). Adherence to anti-TNF therapy in inflammatory bowel diseases: a systematic review. Inflamm Bowel Dis.

[12] Kane S., Huo D., Aikens J. (2003). Medication nonadherence and the outcomes of patients with quiescent ulcerative colitis. Am J Med.

[13] Kane S.V., Chao J., Mulani P.M. (2009). Adherence to infliximab maintenance therapy and health care utilization and costs by Crohn's disease patients. Adv Ther.

[14] van der Have M., Oldenburg B., Kaptein A.A. (2016). Non-adherence to anti-TNF therapy is associated with illness perceptions and clinical outcomes in outpatients with inflammatory bowel disease: results from a prospective multicentre study. J Crohns Colitis.

[15] Severs M., Mangen M.J., Fidder H.H. (2017). Clinical predictors of future nonadherence in inflammatory bowel disease. Inflamm Bowel Dis.

[16] Coenen S., Weyts E., Ballet V. (2016). Identifying predictors of low adherence in patients with inflammatory bowel disease. Eur J Gastroenterol Hepatol.

[17] Wentworth B.J., Buerlein R.C.D., Tuskey A.G. (2018). Nonadherence to biologic therapies in inflammatory bowel disease. Inflamm Bowel Dis.

[18] Shah N.B., Haydek J., Slaughter J. (2020). Risk factors for medication nonadherence to self-injectable biologic therapy in adult patients with inflammatory bowel disease. Inflamm Bowel Dis.

[19] Harris P.A., Taylor R., Thielke R. (2009). Research electronic data capture (REDCap)--a metadata-driven methodology and workflow process for providing translational research informatics support. J Biomed Inform.

[20] Harris P.A., Taylor R., Minor B.L. (2019). The REDCap consortium: building an international community of software platform partners. J Biomed Inform.

[21] Banks A.M., Peter M.E., Holder G.M. (2020). Adherence to disease-modifying therapies at a multiple sclerosis clinic: the role of the specialty pharmacist. J Pharm Pract.

[22] Berger N., Peter M., DeClercq J. (2020). Rheumatoid arthritis medication adherence in a health system specialty pharmacy. Am J Manag Care.

[23] Shah N.B., Mitchell R.E., Proctor S.T. (2019). High rates of medication adherence in patients with pulmonary arterial hypertension: an integrated specialty pharmacy approach. PLoS One.

[24] Tan H., Yu J., Tabby D. (2010). Clinical and economic impact of a specialty care management program among patients with multiple sclerosis: a cohort study. Mult Scler.

[25] Barnes E., Zhao J., Giumenta A. (2020). The effect of an integrated health system specialty pharmacy on HIV antiretroviral therapy adherence, viral suppression, and CD4 count in an outpatient infectious disease clinic. J Manag Care Spec Pharm.

[26] Bagwell A., Kelley T., Carver A. (2017). Advancing patient care through specialty pharmacy services in an academic health system. J Manag Care Spec Pharm.

[27] Alexakis C., Saxena S., Chhaya V. (2018). Smoking status at diagnosis and subsequent smoking cessation: associations with corticosteroid use and intestinal resection in Crohn's disease. Am J Gastroenterol.

[28] Limsrivilai J., Stidham R.W., Govani S.M. (2017). Factors that predict high health care utilization and costs for patients with inflammatory bowel diseases. Clin Gastroenterol Hepatol.

[29] Singh S., George J., Boland B.S. (2018). Primary non-response to tumor necrosis factor antagonists is associated with inferior response to second-line biologics in patients with inflammatory bowel diseases: a systematic review and meta-analysis. J Crohns Colitis.

[30] Feagan B.G., Sandborn W.J., Gasink C. (2016). Ustekinumab as induction and maintenance therapy for Crohn's disease. N Engl J Med.

[31] Sands B.E., Feagan B.G., Rutgeerts P. (2014). Effects of vedolizumab induction therapy for patients with Crohn's disease in whom tumor necrosis factor antagonist treatment failed. Gastroenterology.

[32] Axelrad J.E., Sharma R., Laszkowska M. (2019). Increased healthcare utilization by patients with inflammatory bowel disease covered by Medicaid at a tertiary care center. Inflamm Bowel Dis.

[33] Flasar M.H., Chao J., Ozbay A.B. (2016). Biological and immunomodulator use in Crohn's disease in a Medicaid population. Inflamm Bowel Dis.

[34] Park K.T., Ehrlich O.G., Allen J.I. (2020). The cost of inflammatory bowel disease: an initiative from the Crohn's & Colitis Foundation. Inflamm Bowel Dis.

[35] Regueiro M., Click B., Anderson A. (2018). Reduced unplanned care and disease activity and increased quality of life after patient enrollment in an inflammatory bowel disease medical home. Clin Gastroenterol Hepatol.

[36] Lores T., Goess C., Mikocka-Walus A. (2021). Integrated psychological care reduces health care costs at a hospital-based inflammatory bowel disease service. Clin Gastroenterol Hepatol.

